# *Inquilinus limosus* in Patients with Cystic Fibrosis, Germany

**DOI:** 10.3201/eid1103.041078

**Published:** 2005-03

**Authors:** Nele Wellinghausen, Andreas Essig, Olaf Sommerburg

**Affiliations:** * University of Ulm, Ulm, Germany

**Keywords:** cystic fibrosis, drug-resistant microbes, molecular diagnostic techniques, epidemiology, dispatch

## Abstract

We identified *Inquilinus limosus*, a recently described α-proteobacterium*,* in sputum of 2 patients with cystic fibrosis whose respiratory tracts were persistently colonized for >9 months. We present data on the epidemiology, antimicrobial susceptibility, and molecular characteristics of *I. limosus*.

Chronic microbial colonization of the respiratory tract, leading to exacerbations of pulmonary infection, is the major cause of disease and death in patients with cystic fibrosis (CF). Typical pathogens in respiratory secretions of patients with CF include *Pseudomonas aeruginosa*, *Staphylococcus aureus*, *Haemophilus influenzae*, and *Burkholderia cepacia* complex (*1*–*3*). Other gram-negative glucose nonfermenters, such as *Achromobacter xylosoxidans*, *B. gla­dioli*, *Ralstonia pickettii*, and *Stenotrophomonas maltophilia* are also occasionally reco­vered from respiratory samples from CF patients, but their pathogenic importance remains to be clarified (*2*–*4*).

Determining the clinical relevance of nonfermentative microbes is hampered by the difficulty in identifying these pathogens by conventional laboratory techniques. Recent studies that applied molecular approaches to identify unusual pathogens in patients with CF showed various infrequently encountered and novel species (*4*–*7*). In 1 of these studies, the gram-negative nonfermentative species *Inquilinus limosus* was newly described in respiratory secretions of 8 patients with CF in the United States (*8*). *I. limosus* belongs to the α-proteobacteria and is, thus, not closely related to *B. cepacia* complex or *P. aeruginosa* (*8*).

To detect unusual gram-negative microbes in respiratory samples, we screened all patients attending the CF clinic of the University Hospital, Ulm, Germany, from May 2002 to September 2004 (N = 85 patients). Respiratory samples (N = 459 samples) were plated with a 10-μL loop on sheep blood agar (Heipha, Heidelberg, Germany), MacConkey agar (Heipha), and *B. cepacia* complex selective agar (containing 100 mg/L ticarcillin and 300,000 IU/L polymyxin B, MAST Diagnostica, Reinfeld, Germany). All plates were incubated for 48 h at 36°C under ambient air, and the *B. cepacia* complex selective agar was incubated another 5 days at room temperature. *I. limosus* was recovered from sputum samples of 2 patients (0.9%).

## Case Reports

### Patient 1

Strain A was isolated from a 17-year-old male patient with CF with persistent colonization of the respiratory tract since childhood with *S. aureus*, *P. aeruginosa*, including the mucoid variant, and *H. influenzae*. In the initial sputum sample, apart from ≈10^6^ CFU/mL of *S. aureus*, 10^5^ CFU/mL of mucoid *P. aeruginosa*, and 10^3^ CFU/mL of *Candida albicans*, 10^4^ CFU/mL of a mucoid gram-negative rod was isolated from *B. cepacia* complex selective agar after 6 days of incubation. The isolate had positive oxidase and catalase reaction, but failed to grow on MacConkey agar. It was identified by using Api 20NE as *Sphingomonas paucimobilis* with questionable profile (Code 0427544). Final identification of *I. limosus* was achieved by sequencing the 16S rRNA gene with the primers 16Sfor and 16Srev (*9*) and a Dye Terminator Cycle Sequencing Ready Reaction Kit (Applied Biosystems, Warrington, United Kingdom) on a 310 Genetic Analyser (Abi Prism). The isolate showed 99.8% sequence homology to the 16S rDNA sequence of the *I. limosus* type strain (LMG 20952T) by using the BLAST algorithm.

During sequence analysis, we discovered a mistake in the *I. limosus* type strain 16S rDNA sequence deposited in GenBank (accession no. AY043374): the CGGGTC motif, repeated twice from base 956 to 967 in AY043374, is only found once in the type strain’s 16S rRNA gene, such as in the *Inquilinus* sp. strain AU1979 (accession no. AY043375 [8]) and in our isolates. We performed susceptibility testing by using Etest (VIVA Diagnostics, Solna, Sweden) on Mueller-Hinton agar, incubated at 36°C for 48 h. In addition, susceptibility testing against colistin was performed by disk diffusion with 10-μg disks (BD, Heidelberg, Germany) on Mueller-Hinton agar (McFarland 0.5, 48 h incubation). All results are shown in the Table. At the time of sputum sampling, the patient was clinically well, with normal values of C-reactive protein, leukocytes, and erythrocyte sedimentation rate. He regularly played soccer. A lung function test was not done. Two weeks after his visit, an elective 14-day course of antimicrobial therapy was initiated consisting of intravenous (IV) ceftazidime (3 g 3 times daily) and IV tobramycin (500 mg once daily) because of *P. aeruginosa* colonization.

Five and a half months later, the patient was seen in the CF clinic again. He was still in very good clinical condition. Results of lung function tests conducted 6 weeks after his first visit and at his second visit were the following: vital capacity, 3.27l/3.34l (77%/74% of predicted vital capacity); and forced expiratory volume, 1 s 2.81l/2.89l (79%/77% of predicted forced expiratory volume). Sputum culture showed ≈10^6^ CFU/mL of *S. aureus*, 10^4^ CFU/mL of mucoid *P. aeruginosa*, 10^3^ CFU/mL of *Aspergillus fumigatus*, and again 10^3^ CFU/mL of *I. limosus*. We performed pulsed-field gel electrophoresis (PFGE) of the isolates with the CHEF DRIII equipment (BioRad, Munich,Germany) in 1% agarose at 14°C and a constant voltage of 200 V (*10*), with the restriction enzyme *XbaI* (*11*). Results showed that the strain (A-2) was identical to the former isolate of the same patient (A-1) (Figure). A 14-day course of oral ciprofloxacin (750 mg twice daily) was initiated because of *P.* *aerugi­nosa* colonization.

**Figure:**
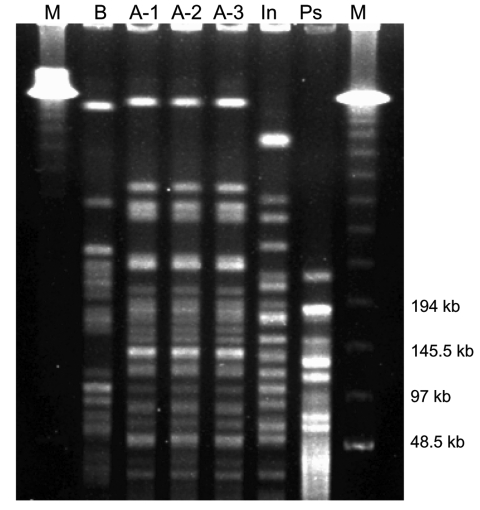
Pulsed-field gel electrophoresis of *Inquilinus limosus*. M, molecular weight marker 48.5 to 970 kbp (BioRad 170-3635); In, *Inquilinus limosus* type strain LMG 20952; Ps, *Pseudomonas aeruginosa* ATCC 27853.

Four months later, the strain was still detected in his sputum. The isolate (A-3) grew in low quantities (10^3^ CFU/mL) and was accompanied by 10^6^ CFU/mL of *S. aureus*, 10^6^ CFU/mL of mucoid *P. aeruginosa*, 10^3^ CFU/mL of *A. fumigatus*, and 10^4^ CFU/mL of *C. albicans*. PFGE showed identity with the former strains (Figure), and antimicrobial susceptibility was unchanged (Table). Lung function test showed a vital capacity of 3.81l (68% of predicted vital capacity) and a forced expiratory volume of 3.25l (71% of predicted forced expiratory volume), and the patient was in good health. More than 2 months later, the *I. limosus* strain was no longer cultured from his sputum, while *P. aeruginosa* (10^3^ CFU/mL), *S. aureus* (10^6^ CFU/mL), and *C. albicans* (10^3^ CFU/mL) were still found.

**Table:** Antimicrobial susceptibility of *Inquilinus limosus* strains

Antimicrobial agent	MIC (µg/mL)				
	Isolate A-1	Isolate A-2	Isolate A-3	Isolate B	LMG 20952^T^
Trimethoprim/sulfamethoxazole	>32	>32	>32	>32	>32
Amikacin	8	32	32	>256	>256
Gentamicin	8	16	12	>256	12
Tobramycin	>256	>256	>256	>256	>256
Ampicillin	>256	>256	>256	>256	>256
Piperacillin/tazobactam	>256	>256	>256	>256	>256
Cefotaxime	>32	>32	>32	>32	>32
Ceftazidime	4	4	4	0.5	32
Imipenem	0.012	0.012	0.016	0.006	0.016
Ciprofloxacin	0.032	0.032	0.032	0.500	0.064
Colistin*	Resistant	Resistant	Resistant	Resistant	Resistant

*Susceptibility testing done by disk diffusion (see text); no zone of inhibition was seen.

### Patient 2

Strain B was isolated from a sputum sample of a 14-year-old female patient with CF with respiratory colonization since childhood with *P. aeruginosa*, including the mucoid variant, and *H. influenzae*. The mucoid isolate of *I. limosus* grew in large quantities (≈10^5^ CFU/mL) on *B. cepacia* complex selective agar after 5 days of incubation. In addition, ≈10^5^ CFU/mL mucoid of *P. aeruginosa*, 10^3^ CFU/mL of *A. fumigatus* and *C. albicans* were found in the sputum sample. Colonies were oxidase- and catalase-positive and failed to grow on MacConkey agar. Api 20NE showed *Sphingo­monas paucimobilis* with questionable profile (Code 0424744), and identification was achieved by 16S rDNA sequencing, as described above. The isolate showed 99.3% sequence homology to the *I. limosus* type strain (LMG 20952^T^). PFGE showed a different band pattern, which suggests that this strain was different from strain A and, thus, excluded cross-infection between both patients (Figure).

The antimicrobial susceptibility of strain B was comparable to that of strain A, apart from higher MIC values for amikacin and gentamicin and lower values for ceftazidime (Table). Like the first patient, this patient was also in good health and active. At the time of sputum sampling, pulmonary function and laboratory tests were not done, but a pulmonary function test conducted 2 weeks before showed a vital capacity of 2.96l (74% of predicted vital capacity) and a forced expiratory volume 1 of 1.95l (77% of predicted forced expiratory volume 1), leukocyte count and erythrocyte sedimentation rate were normal, and C-reactive protein was slightly elevated to 5.8 mg/L. During the following 2 months, 6 sputum samples were investigated, but *Inquilinus* was not detected again, while *P. aeruginosa* was still present in a concentration of 10^5^ CFU/mL. The patient moved to another CF clinic and was lost to follow-up.

## Conclusions

To our knowledge, isolation of *I. limosus* from clinical samples has only been described in 1 study (*8*). The prevalence of this species in our CF clinic between May 2002 and September 2004 was 2.4%. The natural reservoir of *I. limosus* has yet to be discovered, but its relatedness to other nonfermentative rods suggests environmental sources. *Inquilinus* might be overlooked in clinical samples because of its rather slow growth and failure to grow on MacConkey agar. Recovery of *Inquilinus* can be improved by using selective media containing polymyxin B or colistin and ticarcillin, such as *B. cepacia* complex selective agar, and prolonged incubation at 36°C. The necessary duration of incubation has yet to be determined since our isolates grew better at 36°C than at room temperature. Identifying the species is difficult since it is not contained in the databases of commercial identification kits and its mucoid appearance may lead to confusion with mucoid *P. aeruginosa* strains. This species’ failure to grow on MacConkey agar, positive oxidase reaction, and typical antimicrobial susceptibility profile (see below) in respiratory samples of CF patients should arouse suspicion. Identification of *I. limosus* can be confirmed by 16S rRNA gene sequencing (*8*). *I. limosus* is able to persist in the respiratory tract of CF patients for several months. As with *P. aeruginosa*, abundant amounts of mucus with *I. limosus* infection may favor persistence and chronic infection. However, the pathogenic role of *I. limosus* in the patients described here is unclear. The stepwise deterioration of pulmonary function seen in patient 1 may also be attributed to irregular intervals of inhalation and of elective antimicrobial therapy. The patient had finished his education and had started his first employment.

*I. limosus* shows a distinct antimicrobial susceptibility profile with high MICs for cotrimoxazole, most aminoglycosides, ampicillin, cefotaxime, and piperacillin/tazobactam. Although ceftazidime and ciprofloxacin would be interpreted as susceptible applying the NCCLS interpretation criteria for *P. aeruginosa* (*12*), strain A persisted in the respiratory tract of the patient for several months after therapy with these substances. *Inquilinus* may be effectively protected from the action of antimicrobial agents by mucus production, and local host factors may also contribute to colonization and persistency. Further studies are necessary to evaluate the epidemiology and clinical importance of *I. limosus* as well as the therapeutic options in CF patients and in other patient groups. For instance, screening large CF patient groups by selective culture methods or molecular methods, like the use of specific fluorescence in situ hybridization probes or polymerase chain reaction assays, are desirable for assessing the epidemiology of the species. Longitudinal studies of infected patients are valuable in evaluating the clinical relevance and the factors influencing persistency of *Inquilinus*.
